# *Moraea sisyrinchium* inhibits proliferation, cell cycle, and migration of cancerous cells, and decreases angiogenesis in chick chorioallantoic membrane

**DOI:** 10.22038/IJBMS.2023.70554.15353

**Published:** 2024

**Authors:** Roghayeh Rashidi, Ala Montazeri, Mohammad Soukhtanloo, Shirin Ghasemian, Mohammad Sadegh Amiri, Maede Hasanpour, Elham Einafshar, Ahmad Ghorbani

**Affiliations:** 1 Department of Pharmacology, Faculty of Medicine, Mashhad University of Medical Sciences, Mashhad, Iran; 2 Department of Pharmacodynamics and Toxicology, School of Pharmacy, Mashhad University of Medical Sciences, Mashhad, Iran; 3 Department of Clinical Biochemistry, Faculty of Medicine, Mashhad University of Medical Sciences, Mashhad, Iran; 4 Department of Pharmacognosy, School of Pharmacy, Mashhad University of Medical Sciences, Mashhad, Iran; 5 Department of Biology, Payame Noor University, Tehran, Iran; 6 Biotechnology Research Center, Pharmaceutical Technology Institute, Mashhad University of Medical Sciences, Mashhad, Iran; 7 Pharmacological Research Center of Medicinal Plants, Mashhad University of Medical Sciences, Mashhad, Iran

**Keywords:** Glioblastoma, Hepatocellular carcinoma, HepG2, Iridaceae, U87

## Abstract

**Objective(s):**

Experimental studies reported that some plants in the genus of *Moraea* (Iridaceae family) show anticancer potential. This study aimed to evaluate the effects of *Moraea sisyrinchium* on U87 glioblastoma multiforme and HepG2 liver cancer cells.

**Materials and Methods:**

The cells were incubated for 24 hr with hydroalcoholic extract of the stem, flower, and bulb of *M. sisyrinchium*. Then, the cell proliferation (MTT) assay, cell cycle analysis (propidium iodide staining), cell migration test (scratch), Western blotting (Bax and Bcl-2 expression), and gelatin zymography (for matrix metalloproteinases, MMPs) were performed. Oxidative stress was evaluated by determining the levels of reactive oxygen species and lipid peroxidation. Angiogenesis was evaluated on chick embryo chorioallantoic membrane.

**Results:**

The extracts of the flower, stem, and bulb significantly decreased the proliferation of HepG2 and U87 cells. This effect was more for U87 than HepG2 and for the bulb and stem than the flower. In U87 cells, the bulb extract increased oxidative stress, cell cycle arrest, and the Bax/Bcl-2 ratio. Also, this extract suppressed the migration ability of HepG2 and U87 cells, which was associated with the inhibition of MMP2 activity. In addition, it significantly reduced the number and diameter of vessels in the chorioallantoic membrane. Liquid chromatography-mass spectrometry revealed the presence of xanthones (bellidifolin and mangiferin), flavonoids (quercetin and luteolin), isoflavones (iridin and tectorigenin), and phytosterols (e.g., stigmasterol) in the bulb.

**Conclusion:**

*M. sisyrinchium* bulb decreased the proliferation and survival of cancer cells by inducing oxidative stress. It also reduced the migration ability of the cells and inhibited angiogenesis.

## Introduction

Plant-derived compounds are good candidates for finding new anticancer drugs (1, 2). In the last decades, the anticancer activity of many phytochemicals and plant extracts has been proposed by experimental and clinical studies (1, 3, 4). The *in vitro* investigations showed that some secondary metabolites of the plants suppress cancer cells through oxidative DNA damage and induction of apoptosis (3). Also, several phytochemicals have been shown to prevent the invasion of cancer cells through inhibiting matrix metalloproteinases (MMPs) activities and modulating different signaling pathways such as the PI3k/Akt pathway, VEGF pathway, and MAPK pathway (5-7).

Previous studies reported that some plants in the family of *Iridaceae* such as *Moraea polystachya *show cytotoxic activity against cancer cells (8, 9). *Moraea sisyrinchium *(synonym: *Gynandriris sisyrinchium* and *Iris sisyrinchium*) is one of the plants in this family that is distributed in many countries including Egypt, Turkey, Jordan, and Iran. (10-12). The pharmacological effects of this plant have not been well-determined so far. It has been suggested that its leaves and bulbs have antimicrobial activities (13). In an *in vitro* study, Al-Qudah *et al*. showed that isoflavones isolated from this plant reduced the viability of human promyelocytic leukemia cells (12). As far as we know, no other study has examined the anticancer activity of *M. sisyrinchium. *

Hepatocellular carcinoma, the most common type of primary liver cancer, is one of the leading causes of cancer-related deaths in the world. The incidence rate of liver cancers has increased over the last decades and is expected to rise in future years (14). Glioblastoma is one of the most common malignant tumors of the brain and accounts for approximately 15% of all primary brain tumors and 46% of primary malignant brain tumors (15). Despite advances in surgery, radiotherapy, and chemotherapy, the survival of patients with hepatocellular carcinoma and glioblastoma multiform is low (16, 17). Therefore, many studies are designed to find novel anti-cancer substances against these cancers. The present work aimed to examine the potential cytotoxic effect of *M. sisyrinchium *against HepG2 liver cancer and U87 glioblastoma multiform cells.

## Materials and Methods

***Materials***

HepG2, U87, and L929 cell lines were obtained from Pasteur Institute (Tehran, Iran) and maintained at 37° C in a humidified atmosphere containing 5% CO_2_. Doxorubicin and Temozolomide were from Samen Pharmaceutical Company (Mashhad, Iran) and MSD Inc. (Germany), respectively. Sodium citrate, 3-(4,5-dimethylthiazol-2-yl)-2,5-diphenyl tetrazolium (MTT), Triton X-100, 5,5’-dithiobis-(2-nitrobenzoic acid) (DTNB), thiobarbituric acid, dichlorodihydrofluorescein diacetate (H_2_DCF-DA), bicinchoninic acid protein assay kit, and propidium-iodide (PI) were purchased from Sigma-Aldrich (St. Louis, MO, USA). High glucose Dulbecco’s Modified Eagle’s medium (DMEM)‎ and fetal bovine serum (FBS) were purchased from Gibco (Grand Island, NY, USA). Polyvinylidene difluoride membrane was obtained from GE Healthcare Life Sciences (Germany). Primary antibodies (Bax, Bcl-2, β-actin) and secondary antibody (horseradish peroxidase-conjugated goat anti-rabbit IgG) were purchased from Abcam (Cambridge, MA, USA).

***Preparation of M. sisyrinchium extract***

*M. sisyrinchium *was collected from Dargaz mountains (Northeast Iran) and identified by Dr. Mohammad Sadegh Amiri at the herbarium of Dargaz Payame Noor University (voucher specimen number: 712). The flowers and stems were dried in the shade and ground separately to a fine powder. The bulb part of the plant was kept in the freezer after collection until extraction. To prepare the hydroalcoholic extract, a sample (10 gr) of each part of the plant was soaked in 70% ethanol (200 ml) for 3 days at 40 °C with occasional shaking. The obtained extracts were filtered (pore size 250 μm) and then centrifuged for 5 min at 500 *g* to remove sediment particles. The supernatants were dried in an oven at 40 °C and the dried extracts were kept frozen at less than -18° C until use. The solid residue of the extracts of the flower, stem, and bulb were 40%, 18%, and 5%, respectively.

***Cell culture and treatment***

The HepG2, U87, and L929 (mouse fibroblast as nonmalignant control) cells were cultured in DMEM supplemented with 10% FBS and 1% penicillin/streptomycin solution. For proliferation assay and reactive oxygen species (ROS) evaluation, the cells were seeded in 96-well plates (5×10^3 ^cells/well) and then treated for 24 hr with the extracts (25-400 µg/ml). For cell cycle analysis, the cells were seeded in 12-well plates (5×10^5 ^cells/well) and treated for 24 hr with the extracts. Doxorubicin (0.375-6 µg/ml) and temozolomide (0.75-24 µg/ml) were used as positive controls for HepG2 and U87 cells, respectively.

***Cell proliferation assay***

The cell proliferation was determined using the MTT reagent, which is reduced to purple formazan crystals in the mitochondria of living cells (18). At the end of the treatment time (24 or 48 hr), the medium of each well was removed from the culture plate and fresh DMEM containing MTT reagent (0.05%) was added to the plate. The cells were incubated for 4 hr in a cell culture incubator. Then, the medium was replaced by dimethyl sulfoxide (200 µl per well) to dissolve the formazan crystals. After 20 min, the optical absorbance of each well was measured at 570 and 620 nm (background) using a plate reader (Stat FAX – 2100, Awareness Technology Inc., USA).

***Cell cycle analysis***

After incubation with the extracts (24 hr), the U87 cells were trypsinized, centrifuged (5 min at 500 g), and re-suspended in a buffer containing propidium iodide reagent. This buffer also consisted of 0.1% sodium citrate and 0.1% Triton-X 100, which makes the cell membrane permeable to stain nucleic acids. After 30 min, the cell cycle was evaluated using a FACSCALIBUR™ flow cytometer (Becton Dickinson, Mountain View, CA, USA). The analysis of flow cytometry data was performed by FlowJo ® vX.0.7 software (Tree Star, Ashland, OR, USA). 

***Measurement of intracellular reactive oxygen species (ROS)***

The level of ROS in U87 cells was determined using H_2_DCF-DA, which is converted to highly fluorescent dichlorofluorescein in the presence of ROS. At the end of treatment with the plant extracts (24 hr), the cells were washed with PBS and incubated for 30 min with 20 μM of H2DCF-DA at 37 °C. Then, the fluorescence intensity was determined using an FLUO-star galaxy fluorescence plate reader (Perkin Elmer 2030, Multilabel reader, Finland) at an excitation/emission wavelength of 485/530 nm.

***Lipid peroxidation assay***

Lipid peroxidation in U87 cells was evaluated by determining the malondialdehyde level, the end-product of the peroxidation reaction. After treatment with the plant extracts (24 hr), the cells were trypsinized and centrifuged for 5 min at 500 *g*. Then, the cells were homogenized in an ice-cold KCl solution (1.15%) and centrifuged for 30 min at 2000 *g* (4 °C). Finally, 500 µl of the supernatant was incubated with 500 μl of trichloroacetic acid (15%) and 800 μl of thiobarbituric acid (0.7%). The mixture was boiled in the water bath for 60 min and after cooling centrifuged for 10 min in 1000 *g* (4 °C). The fluorescence intensity of the supernatant was determined at an excitation/emission of 530/550 nm.

***Scratch assay***

The migration capability of HepG2 and U87 cells was evaluated by scratch wound healing assay, which determines their expansion rate on culture surfaces. First, the cells were cultured in 12-well flat-bottom plates until grown to a confluence cell monolayer (approximately 48 hr). Then, a linear scratch was applied in the monolayer using a sterile pipette tip and the cellular debris was washed with PBS. The bulb extract (1-6 μg/ml) was added to the cell culture medium and the incubation continued for 48 hr (37 °C, 5% CO_2_). The experiment was performed in triplicate and images taken from the cells were analyzed using the Image J software package.

***Gelatin zymography***

The activities of MMP-2 and MMP-9 were evaluated by the gelatin zymography method. The U87 cells were seeded in 12-well plates (2 × 10^5^ cells/well) and treated with the bulb extract for 24 hr. Then, the media of each well was centrifuged at 300 *g* for 10 min and 30 μl of the supernatant was electrophoresed on a 10% polyacrylamide gel containing gelatin as substrate (10 mg/ml). The gel was washed with 2% Triton X-100 and incubated in a developing buffer (50 mM Tris-HCl, 5 mM CaCl_2_, 0.2 M NaCl, and 1.0 μM ZnCl2) for 36 hr at 37 °C. After incubation, the gel was stained with Coomassie Brilliant Blue R-250 and then destained using a solution composed of 25% ethanol and 10% acetic acid in dH_2_O. Finally, the gel was photographed by a GS-800 calibrated densitometer (Bio-Rad, HC, USA) and analyzed using the Image J software package.

***Western blot analysis***

The U87 cells were treated for 24 hr with* M. sisyrinchium *bulb extract. Then, they were lysed using RIPA buffer and centrifuged for 10 min at 2500 *g*. The supernatants were subjected to the determination of protein concentration by a bicinchoninic acid protein assay kit. The proteins were separated by 7.5–15% SDS-PAGE and transferred onto a polyvinylidene difluoride membrane. After blocking, the membrane was incubated for 3 hr with primary antibodies against Bax, Bcl-2, and β-actin proteins. Then, the horseradish peroxidase-conjugated secondary antibody was added and the protein bands were made visible using an enhanced chemiluminescence system. The relative expression of Bax and Bcl-2 was determined using the Image J software package.

***Angiogenesis assay***

The effect of the bulb extract on angiogenesis was evaluated using the chicken chorioallantoic membrane model. The fertilized chicken eggs were put in an incubator with a temperature of 37 °C and humidity of 70%. After 8 days, a window (1.5–2 cm) was opened on each eggshell and the extract (25 or 50 µg/egg) or PBS (50 µl, as control) was injected into the chorioallantoic sac. The windows were closed with sterile parafilm and the eggs were returned to the incubator. On day 12, the chorioallantoic membrane vasculatures were photographed using a stereo microscope equipped with a Canon EOS 40D digital camera (Tokyo, Japan). Analysis of the diameter of vessels was performed by Image J software.

***Liquid chromatography-mass spectrometry***

High-performance liquid chromatography-mass spectrometry (LC-MS) was performed in an Agilent 1200 series liquid chromatography coupled with Agilent 6410 triple quadrupole Mass Spectrometer. Liquid chromatography separation was performed on an Agilent Eclipse Plus C18 (2.1×100 mm×3.5 μm) column. The flow rate was set at 0.4 ml/min and the mobile phase consisted of (A) water + 0.1% formic acid and (B) methanol + 0.1% formic acid. The gradient programs were as follows: 0.0-1.0 min 10% B, 1.0-40 min from 10% to 100% (B), 40.0-42.0 min 100% (B), and 42.0-50 min from 100% to 10% (B). The mass spectra were acquired in a range of 100 to 1000 within the 50 min scan time. The positive electrospray ionization (ESI) mode was applied for the mass spectrometer. Mass feature extraction of the acquired LC-MS data and maximum detection of peaks was done using the MZmine analysis software package, version 2.3. 

***Statistical analysis***

Comparison of data between control and concentrations of the plant extracts or reference drugs (doxorubicin and temozolomide) was made by one-way analysis of variance (ANOVA) followed by the Dunnett *post hoc* test. The *P*<0.05 was considered statistically significant and results are presented as mean ± SEM.

## Results

***Effects of M. sisyrinchium on cell proliferation***

**Figure 1:**
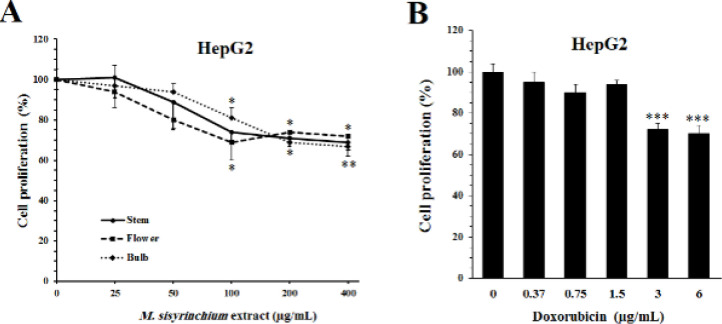
Effects of *Moraea sisyrinchium* (A) and doxorubicin (B) on the proliferation of liver cancer (HepG2) cells

As shown in Figure 1, the extracts of the flower, stem, and bulb of *M. sisyrinchium *at concentrations of ≥ 100 µg/ml significantly decreased the proliferation of HepG2 cells after 24 hr (*P*<0.05). Doxorubicin as a reference drug showed antiproliferative activity at much lower concentrations than the plant extracts (3 and 6 µg/ml, *P*<0.001).

**Figure 2:**
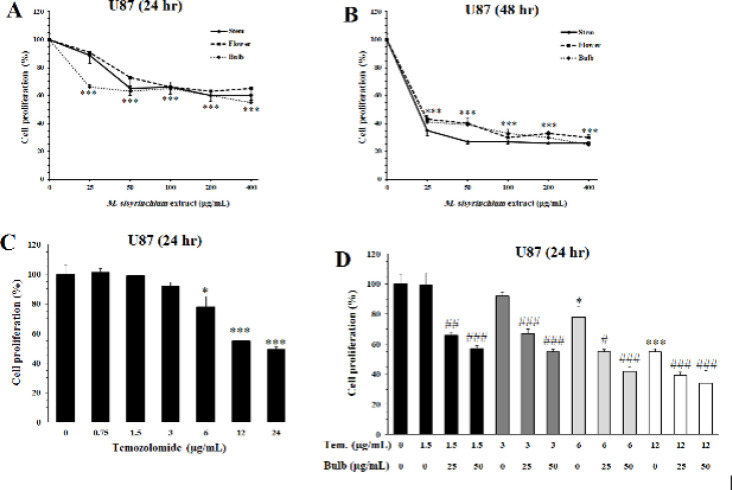
Effects of *Moraea sisyrinchium *and temozolomide (Tem) on the proliferation of glioblastoma multiforme (U87) cells

Similarly, incubation of U87 cells for 24 hr with the extracts of flower, stem, or bulb of *M. sisyrinchium *significantly decreased their proliferation (*P*<0.001, at ≥ 25 µg/ml) (Figure 2). The antiproliferative effect of all extracts was more pronounced after 48 hr incubation and the proliferation rate decreased more than 50% (*P*<0.001). However, the decrease in cell proliferation was not concentration-dependent. Temozolomide, as a reference drug for U87 cells, inhibited proliferation at concentrations of ≥ 6 µg/ml (*P*<0.05). The effect of temozolomide at concentrations of 12 and 24 µg/ml was approximately comparable to the effect of bulb extract at 25 µg/ml. The extract of the bulb at concentrations of 25 and 50 µg/ml could increase the antiproliferative activity of temozolomide (1.5–12 µg/ml).

**Figure 3:**
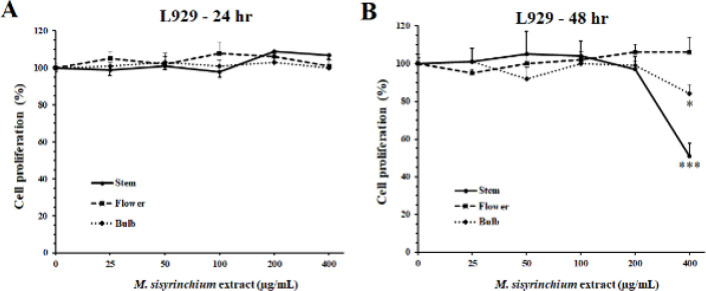
Effects of *Moraea sisyrinchium* on the proliferation of mouse non-malignant fibroblasts (L929)

The tested extracts had no antiproliferative effect on nonmalignant L929 cells after 24 hr. However, after 48 hr, the extracts of stem and bulb decreased L929 proliferation at 400 µg/ml (*P*<0.05, Figure 3). The IC_50_ values of the extracts of *M. sisyrinchium *for HepG2, U87, and L929 cell lines are shown in Table 1.

***Effects of M. sisyrinchium on oxidative stress***

**Figure 4:**
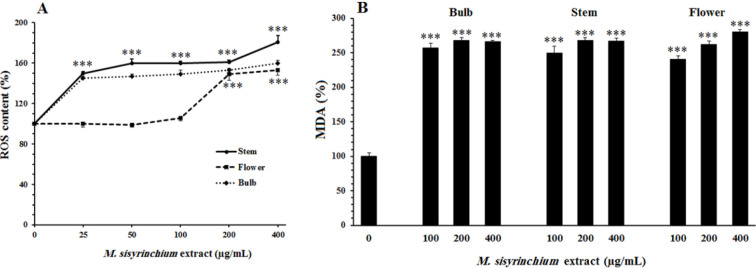
Effects of *Moraea sisyrinchium* on reactive oxygen species (ROS) (A) and malondialdehyde (B) content in glioblastoma multiforme (U87) cells

Incubation of U87 cells for 24 hr with the extract of stem or bulb significantly (*P*<0.001) increased the ROS content (at > 25 µg/ml, Figure 4A). However, the extract of flower could increase the ROS level only at high concentrations (200 µg/ml and 400 µg/ml, *P*<0.001).

An increase in ROS content can lead to peroxidation of cellular lipids, which can be assessed by evaluating the level of malondialdehyde. As shown in Figure 4B, all the tested extracts significantly increased the level of malondialdehyde in U87 cells (*P*<0.001).

***Effects of M. sisyrinchium on sub-G1 cell population***

**Figure 5:**
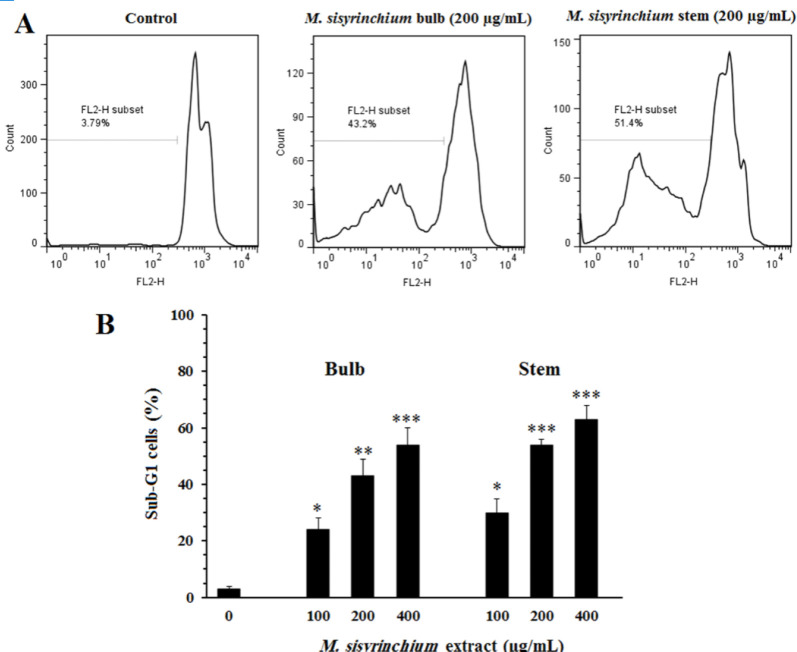
Effects of *Moraea sisyrinchium *on the cell cycle of glioblastoma multiforme (U87) cells

As shown in Figure 5, the culture of U87 cells in the presence of extracts of stem or bulb significantly increased the percentage of cells in the sub-G1 stage compared to untreated cells (*P*<0.05). 

***Effects of M. sisyrinchium on cell migration***

**Figure 6:**
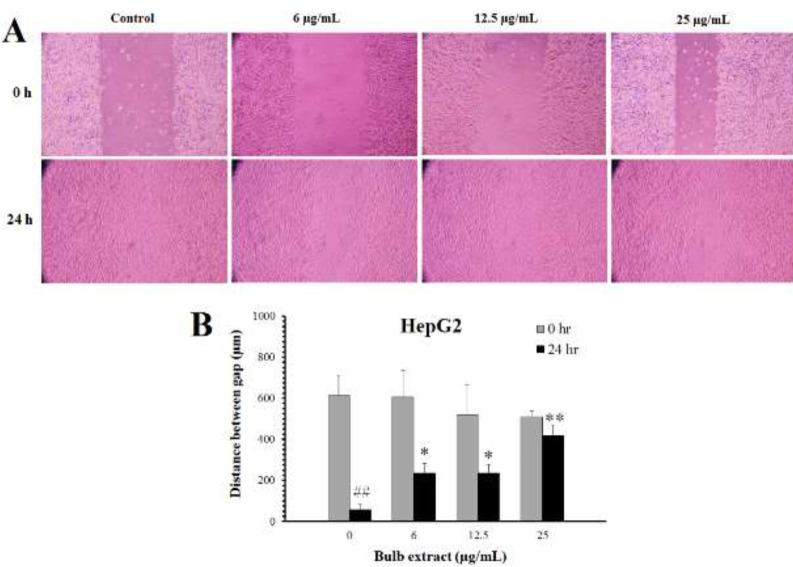
Effects of *Moraea sisyrinchium* bulb on the migration of liver cancer (HepG2) cells

**Figure 7:**
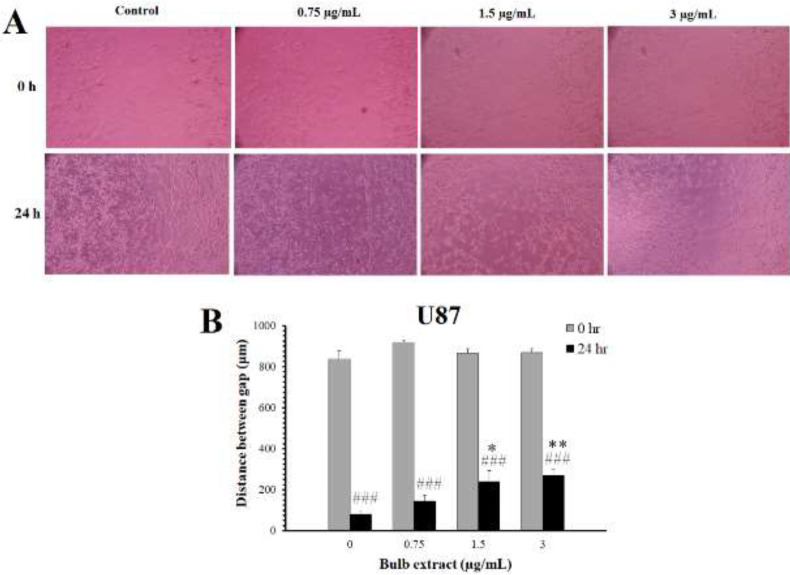
Effects of *Moraea sisyrinchium* bulb extract on the migration of glioblastoma multiforme (U87) cells

Representative photographs of HepG2 cells treated with non-cytotoxic concentrations of the bulb extract are shown in Figure 6. Scratch migration assay showed that the distance between the cells disappeared after 24 hr in the untreated control group. The extract of the bulb at concentrations of 6, 12.5, and 25 µg/ml significantly decreased the migration rate (*P*<0.05). Similarly, this extract significantly (*P*<0.05) inhibited the migration of U87 cells at non-toxic concentrations (1.5 µg/ml and 3 µg/ml, Figure 7).

***Effect of M. sisyrinchium on the activity of MMPs***

**Figure 8:**
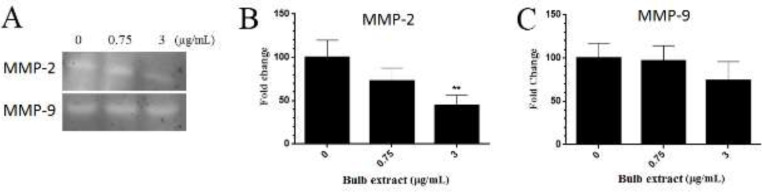
Effects of *Moraea sisyrinchium* bulb extract on the activity of matrix metalloproteinases (MMPs) in U87 cells

As shown in Figure 8, the activity of MMP-2 in U87 cells significantly decreased in the presence of 3 µg/ml of the bulb extract (*P*<0.01). However, treatment with this extract had no significant effect on the MMP-9 activity.

***Effect of M. sisyrinchium on bax and Bcl-2 expression***

**Figure 9:**
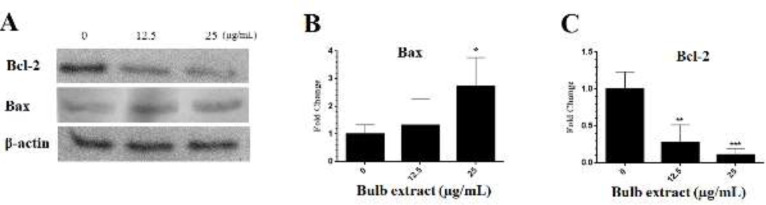
Effects of *Moraea sisyrinchium *on the expression of pro-apoptotic (Bax) and anti-apoptotic (Bcl-2) proteins in U87 cells

The analysis of western blotting showed that the extract of the bulb at a concentration of 25 μg/ml significantly increased the expression of Bax protein in U87 cells (*P*<0.05, Figure 9). On the other hand, the expression of Bcl-2 was down-regulated at concentrations of 12.5 and 25 μg/ml of the extract (*P*<0.01).

***Effect of M. sisyrinchium on angiogenesis***

**Figure 10:**
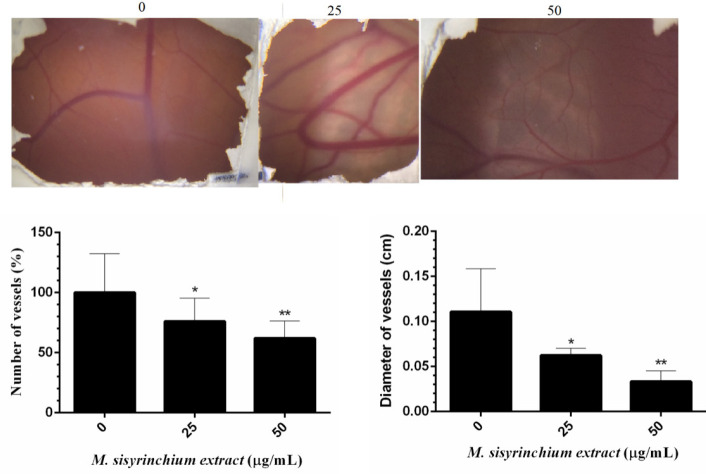
Effects of *Moraea sisyrinchium* bulb extract on angiogenesis in a chicken chorioallantoic membrane model

The eggs that were treated with 25 or 50 µg of the bulb extract had fewer vessels in their chorioallantoic membrane than the control group (*P*<0.05, Figure 10). Also, the diameter of vessels formed in the membrane significantly decreased in groups treated with this extract (*P*<0.05).

***LC-MS analysis of M. sisyrinchium hydroalcoholic extract***

**Figure 11:**
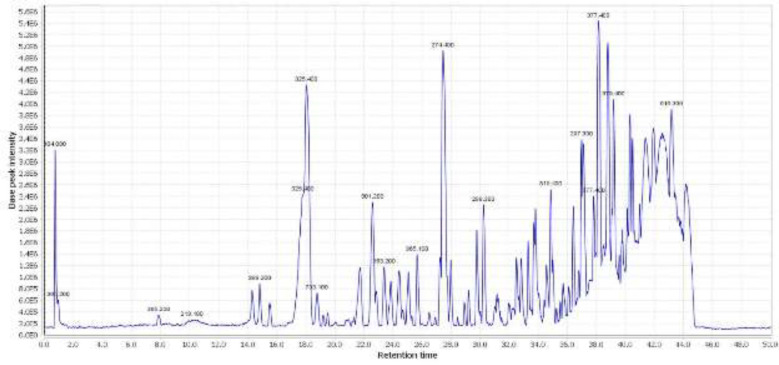
Total ion chromatogram of *Moraea sisyrinchium *bulb extract

The LC-MS analysis showed at least 31 compounds in the hydroalcoholic extract of the *M. sisyrinchium *bulb (Table 2). The total ion chromatogram of the extract is shown in Figure 11. The MS spectral data were compared with the reported compounds in some previous literature. Some examples of extracted ion chromatograms from the total ion chromatogram and its related mass are shown in Supplementary Figure S1-S5. Most of the compounds detected in the hydroalcoholic extract have been previously reported in *Iris* species. Some xanthones including iriflophenone 4-O-hexoside, mangiferin, 7-O-methyl-mangiferin, iriflophenone 4-O-(6”-acetyl)-hexoside, polygalaxanthone, bellidifolin, and iriflophenone were detected in the extract. Some flavonoids including luteolin 8-hexoside, apigenin 6-C-hexoside, quercetin 3-O-glucoside, and irisdichotin B were also identified. The compounds tectoridin, iristectorin B and A, iristectorigenin A-O-hexuronide, iridin, irifloside, tectorigenin, iristectorigenin A, irigenin, irilone, iriflogenin, and irisolidone were characterized as isoflavones. Therefore, it seems that bulb extract is a good source of xanthones, flavonoids, and isoflavones.

## Discussion

The present study showed that different parts of *M. sisyrinchium *decrease the proliferation and migration of cancerous cells *in vitro*. The anticancer effect of the stem and bulb was higher than the flower for both HepG2 and U87 cells. Although the effect of *M. sisyrinchium *on cell proliferation was time-dependent, it was not concentration-dependent. Since the extract of stem considerably decreased the proliferation of non-malignant L929 control cells at high concentrations, we focused on the bulb extract (which seems to be safer) for the next experiments. This extract showed an IC_50_ value of 20 μg/ml for U87 cells after 48 hr incubation, which is within the acceptable range (IC_50_ < 30 μg/ml for the crude extracts) specified by the American National Cancer Institute. For cell cycle evaluation and oxidative stress assessment, we mainly focused on U87 cells because the effect of bulb extract (at 25 µg/ml) was almost comparable to that of temozolomide (at 24 µg/ml), while this extract failed to show antiproliferative activity on HepG2 at concentrations close to that of doxorubicin.

Doxorubicin, an antibiotic-derived compound, is used for the treatment of different cancers. The mechanisms responsible for its anticancer effect include disruption of topoisomerase-II-mediated DNA repair and increase of free radicals, which damage cellular membranes, proteins, and DNA (19). In the present study, doxorubicin was used as a reference drug for HepG2 cells and showed antiproliferative activity. A similar activity was observed for temozolomide, as a reference drug against U87 cells. The bulb extract could increase the antiproliferative activity of temozolomide even at concentrations of this drug that did not have a significant action. Therefore, this extract may be a candidate to be an add-on therapy with other medications for glioblastoma. The extract also enhanced the generation of ROS and lipid peroxidation in U87 cells. These effects were associated with an increase in the percentage of sub-G1 cells, suggesting the occurrence of cell cycle arrest or apoptosis due to oxidative stress. In support of this possibility, the bulb extract increased the expression of the pro-apoptotic protein Bax and decreased the anti-apoptotic protein Bcl-2. Although in the present study the extracts increased ROS production and lipid peroxidation in U87 cells, Al-Qudah et al. (12) reported that some isoflavones of this plant act as free-radical scavengers. The reason for this discrepancy may be that in our study the crude extract was used, but in the work of Al-Qudah *et al*., the anti-oxidant effect of isolated isoflavones was investigated.

One of the limitations of our work is that the signaling pathway of apoptosis was not examined in detail. Evaluating the level of other pro-apoptotic molecules including caspases can clarify the effect of *M. sisyrinchium *on this pathway.

The migration of malignant cells to adjacent tissue and distant organs is a major challenge in cancer treatment (20). In the present work, we tested whether *M. sisyrinchium *could decrease the migration capability of glioblastoma and liver cancer cells. The bulb extract at non-cytotoxic concentrations decreased the migration ability of both HepG2 and U87 cells. This effect was accompanied by a decrease in the activity of the MMP-2 enzyme. MMP-2 was shown to modulate glioma cell migration by disrupting the composition of the extracellular matrix and altering the expression of cell surface adhesion receptors (21). Also, the MMP-2 can regulate vascular growth and therefore survival and invasion of glioblastoma (22). We observed that the bulb extract inhibited vascular growth in chick embryo chorioallantoic membrane.

Some types of plant-derived compounds including polyphenols, alkaloids, and terpenes have been found to inhibit cancer initiation and invasion (1, 5). Only limited works have been done to characterize active compounds in *M. sisyrinchium. *Researchers found some flavonoids including apigenin, luteolin, orientin, saponarin, and isovitexin in this plant (11). A study reported that *M. sisyrinchium* contains β-sitosterol, hispidulin, galangustin, ursolic acid, and ladanetin (13). In the present study, LC-MS analysis revealed the presence of several compounds including xanthones (e.g., bellidifolin and mangiferin), flavonoids (e.g., quercetin and luteolin), isoflavones (e.g., iridin and tectorigenin), and phytosterols (e.g., stigmasterol) in the bulb extract. Although it is currently not possible to suggest exactly which compound is responsible for the anticancer effects of *M. sisyrinchium*, previous studies have shown cytotoxic activity for tectorigenin, stigmasterol, mangiferin, tectoridin, iristectorin B, and bellidifolin against different cancerous cells including glioblastoma (tectorigenin) and liver cancer (tectorigenin, stigmasterol) (23-25). Therefore, further studies are needed to determine the exact compound/compounds responsible for the anticancer effect of this plant. For example, it is suggested to prepare different fractions from the crude extract of *M. sisyrinchium* and evaluate the anticancer effect of each fraction.

The cells were incubated for 24 hr. Data are presented as mean ± SEM (n = 4). *P<0.05, **P<0.0.01, and ***P<0.001 compared to untreated cells (concentration of 0).

The cells were incubated for 24 hr or 48 hr with hydroalcoholic extract of the stem, flower, and bulb of* Moraea sisyrinchium *(A and B), temozolomide (C), or a combination of the bulb extract and Tem (D). Data are presented as mean ± SEM (n = 4). *P<0.05, ***P<0.001 compared to untreated cells (concentration of 0); #*P*<0.05, ##*P*<0.01, and ###*P*<0.001 compared to only Tem-treated cells (corresponding concentration).

The cells were incubated for 24 hr (A) or 48 hr (B) with hydroalcoholic extract of the stem, flower, and bulb of *Moraea sisyrinchium.* Data are presented as mean ± SEM (n = 4). *P<0.05 and ***P<0.001 compared to untreated cells (concentration of 0).

**Table 1:** IC_50 _values of hydroalcoholic extracts of different parts of *Moraea sisyrinchium* for liver cancer (HepG2), glioblastoma multiforme (U87), and mouse fibroblast (L929) cell lines

Cell line	Incubation time	Parts of the plant		
		Stem	Flower	Bulb
HepG2	24 hr	596 µg/ml	607 µg/ml	596 µg/ml
U87	24 hr	451 µg/ml	473 µg/ml	418 µg/ml
U87	48 hr	17 µg/ml	22 µg/ml	20 µg/ml
L929	24 hr	No cytotoxicity	No cytotoxicity	No cytotoxicity
L929	48 hr	> 400 µg/ml	No cytotoxicity	> 400 µg/ml

The cells were incubated for 24 hr with hydroalcoholic extract of the stem, flower, and bulb of Moraea sisyrinchium. Data are presented as mean ± SEM (n = 6). ***P<0.001 compared to untreated cells (concentration of 0).

A: Representative histogram of propidium iodide-stained cells treated for 24 hr with hydroalcoholic extract of the bulb or stem of Moraea sisyrinchium. B: Quantitative analysis of cell population in the sub-G1 stage following treatment with the extracts. Data are presented as mean ± SEM (n = 3). *P<0.05, **P<0.01, and ***P<0.001 compared to untreated cells (concentration of 0).

A: Representing photomicrographs of the cells immediately and 24 hr following culture. B: Quantitative analysis of the distance between scratched gap areas. Data are presented as mean ± SEM (n = 3). ##*P*<0.01 compared to time 0 (corresponding concentration); *P<0.05 and **P<0.01 compared to untreated cells (concentration of 0) at time 24 hr

A: Representing photomicrographs of the cells immediately and 24 hr following culture. B: Quantitative analysis of the distance between scratched gap areas. Data are presented as mean ± SEM (n = 3). ###*P*<0.001 compared to time 0 (corresponding concentration); *P<0.05 and **P<0.01 compared to untreated cells (concentration of 0) at time 24 hr.

Extracellular activities of MMP-2 and MMP-9 were evaluated by the gelatin zymography method. The data are presented as the mean ± SEM (n = 3). **P<0.01 compared to untreated cells (concentration of 0).

The cells were treated with the bulb extract for 24 hr and then subjected to western blot analysis. The data are presented as the mean ± SEM (n = 3). *P<0.05, **P<0.01, and ***P<0.001 compared to untreated cells (concentration of 0).

Representative photographs of the chorioallantoic membrane are shown for control and treated eggs (A). B & C: Quantitative analysis for the number and diameter of vessels. The data are presented as the mean ± SEM (n = 3). *P<0.05 and **P<0.01 compared to untreated cells (concentration of 0).

**Table 2:** Peak assignment of phytochemicals in the hydroalcoholic extract of *Moraea sisyrinchium* bulb using LC-MS analysis in the positive mode

Peak No.	Compound	t_R_ (min)	[M+1] (*m/z*)	Ref.
1	Iriflophenone 4-O-hexoside	31.0	409.3	(21, 22)
2	Mangiferin	40.1	423.3	(21, 23, 24)
3	7-O-Methyl-mangiferin	39.8	437.3	(21, 22)
4	Iriflophenone 4-O-(6"-acetyl)-hexoside	41.3	451.3	(21, 22)
5	Polygalaxanthone III	39.6	569.2	(21, 22)
6	Bellidifolin	27.5	274.4	(21, 22, 25)
7	Luteolin 8-hexoside	40.9	449.3	(21, 22)
8	Apigenin 6-C-hexoside	43.8	433.3	(21, 22)
9	4'-O-Methyl-apigenin-Chexoside	40.1	447.3	(21, 22)
10	Quercetin 3-O-glucoside	41.0	465.3	(21, 22, 25)
11	Irisdichotin B	40.9	495.3	(21, 22)
12	Tectoridin	40.3	463.2	(21, 22)
13	Iristectorin B	42.0	493.2	(21, 22)
14	Iristectorigenin A 7-O-hexuronide	37.1	507.2	(21, 22)
15	Iristectorin A	42.1	493.3	(21, 22, 25)
16	Iridin	37.1	523.1	(21, 22, 25)
17	7-O-Methyl-tectorigenin 4′-O-(6"-hexosyl)-hexoside	42.0	639.2	(21, 22)
18	Irifloside	34.4	491.1	(21, 22)
19	Irisolidone 7-O-hexoside	41.7	477.3	(21, 22)
20	Tectorigenin	22.6	3012	(21, 22, 25)
21	Dichotomitin 3'-O-hexoside	43.0	521.2	(21, 22)
22	Irilone 4'-O-[6"-(3-hydroxy-3-methylglutaryl)]-hexoside	44.2	605.2	(21, 22)
23	Iristectorigenin A	41.3	331.4	(21, 25)
24	Irigenin	25.3	361.2	(21, 22)
25	Irilone	30.2	299.3	(21, 25)
26	Iriflogenin	40.5	329.4	(21, 25)
27	Irisolidone	34.0	315.3	(21, 25)
29	Iriflophenone	14.4	247.1	(21, 25)
30	Pallasone B Dihydroirisquinone	38.1	377.4	(25)
31	Stigmasterol	40.5	413.3	(25)
32	Unknown	25.7	365.1	-
33	Unknown	18.1	325.4	-
34	Unknown	34.9	319.4	-
35	Unknown	38.8	353.4	-
36	Unknown	43.2	615.3	-

## Conclusion

The extracts of the bulb, stem, and flower of *M. sisyrinchium* inhibited the proliferation and cell cycle of cancerous cells by inducing oxidative stress. The bulb extract also inhibited angiogenesis and the migration capability of the cells by reducing MMP-2 activity. Therefore, *M. sisyrinchium *may be a candidate for complementary treatment of glioblastoma in future studies.

## Authors’ Contributions

A G designed and supervised the study, discussed the results, and wrote the manuscript; R R performed cell culture, treatment, and assays, and wrote the manuscript; A M helped to do the cell culture; M S supervised gelatin zymography; S G performed primary analysis of the plant phytochemicals (HPLC); M S A collected and identified the plant materials; M H and E E supervised and analyzed LC-MS; All authors approved the final version of the manuscript.

## Ethical Approval

The methodology for this study was approved by the Research Ethics Committee of Mashhad University of Medical Sciences (Ethics approval number: IR.MUMS.MEDICAL.REC.1398.418).

## Conflicts of Interest

The authors report no conflicts of interest.
